# LT-K63 Enhances B Cell Activation and Survival Factors in Neonatal Mice That Translates Into Long-Lived Humoral Immunity

**DOI:** 10.3389/fimmu.2020.527310

**Published:** 2020-10-23

**Authors:** Audur Anna Aradottir Pind, Jenny Lorena Molina Estupiñan, Gudbjorg Julia Magnusdottir, Giuseppe Del Giudice, Ingileif Jonsdottir, Stefania P. Bjarnarson

**Affiliations:** ^1^Department of Immunology, Landspitali, The National University Hospital of Iceland, Reykjavik, Iceland; ^2^Faculty of Medicine, School of Health Sciences, University of Iceland, Reykjavik, Iceland; ^3^GSK Vaccines, Siena, Italy

**Keywords:** neonatal vaccination, adjuvant, B cell subsets, BAFF-R, BCMA, plasma cell survival, APRIL (TNFSF13), BAFF - B-cell activating factor

## Abstract

Adjuvants enhance magnitude and duration of immune responses induced by vaccines. In this study we assessed in neonatal mice if and how the adjuvant LT-K63 given with a pneumococcal conjugate vaccine, Pnc1-TT, could affect the expression of tumor necrosis factor receptor (TNF-R) superfamily members, known to be involved in the initiation and maintenance of antibody responses; B cell activating factor receptor (BAFF-R) and B cell maturation antigen (BCMA) and their ligands, BAFF, and a proliferation inducing ligand (APRIL). Initially we assessed the maturation status of different B cell populations and their expression of BAFF-R and BCMA. Neonatal mice had dramatically fewer B cells than adult mice and the composition of different subsets within the B cell pool differed greatly. Proportionally newly formed B cells were most abundant, but they had diminished BAFF-R expression which could explain low proportions of marginal zone and follicular B cells observed. Limited BCMA expression was also detected in neonatal pre-plasmablasts/plasmablasts. LT-K63 enhanced vaccine-induced BAFF-R expression in splenic marginal zone, follicular and newly formed B cells, leading to increased plasmablast/plasma cells, and their enhanced expression of BCMA in spleen and bone marrow. Additionally, the induction of BAFF and APRIL expression occurred early in neonatal mice immunized with Pnc1-TT either with or without LT-K63. However, BAFF^+^ and APRIL^+^ cells in spleens were maintained at a higher level in mice that received the adjuvant. Furthermore, the early increase of APRIL^+^ cells in bone marrow was more profound in mice immunized with vaccine and adjuvant. Finally, we assessed, for the first time in neonatal mice, accessory cells of the plasma cell niche in bone marrow and their secretion of APRIL. We found that LT-K63 enhanced the frequency and APRIL expression of eosinophils, macrophages, and megakaryocytes, which likely contributed to plasma cell survival, even though APRIL^+^ cells showed a fast decline. All this was associated with enhanced, sustained vaccine-specific antibody-secreting cells in bone marrow and persisting vaccine-specific serum antibodies. Our study sheds light on the mechanisms behind the adjuvanticity of LT-K63 and identifies molecular pathways that should be triggered by vaccine adjuvants to induce sustained humoral immunity in early life.

## Introduction

Despite great advances in medical technology in recent years, more than 5 million children under 5 years of age die each year, mostly in developing countries (1). Around half of these deaths occur in the first month of life (1). Vaccines against infectious diseases prevent 2–3 million deaths annually (2). Even though vaccines against many infectious agents are available, antibody (Ab) immunity to many vaccines, including protein conjugated polysaccharide vaccines, in young children wanes over time (3). The low and transient vaccine-induced serum Ab levels in early life are associated with limited activation of germinal centers and decreased survival of plasma cells (4, 5). In germinal centers, B cells go through clonal expansion, affinity maturation and class-switching, and subsequently they can differentiate into Ab-producing plasmablasts and plasma cells or memory cells. Tumor necrosis factor receptor (TNF-R) superfamily members B cell activating factor receptor (BAFF-R), B cell maturation antigen (BCMA), and activator and cyclophilin ligand interactor (TACI) and their ligands; B cell activating factor (BAFF) and a proliferation inducing ligand (APRIL), have been shown to be important for the initiation and maintenance of Ab responses. BAFF-R binds BAFF and is involved in germinal center induction and selection and survival of B cells (6). The development and maintenance of follicular and marginal zone B cells is strongly dependent on BAFF and BAFF-R (7, 8). Both BAFF-R and TACI can induce class switch recombination in B cells (9, 10). BCMA binds BAFF and APRIL, yet APRIL with a higher affinity, and promotes the survival of plasma cells (6, 11). Data on their expression in relation to vaccine responses in early life is limited (12, 13).

Immunological memory is one of the key features of adaptive immunity and the basis of successful vaccination. Recently, it has become clear that the bone marrow is a major resting place for immunological memory [reviewed in (14)]. Memory lymphocytes reside in specialized stromal niches where they rest in terms of proliferation and DNA synthesis and receive the necessary cytokine signals for survival and longevity (15–19). The plasma cell bone marrow survival niche is formed by both static and dynamic components. Reticular stromal cells form direct contacts with sessile plasma cells and are the main organizers of the niche structure (20) while hematopoietic accessory cells of the niche are proliferating and accumulate in close proximity of the plasma cells (21).

Several hematopoietic cell types have been reported to support plasma cell longevity. In mice, eosinophils, megakaryocytes, and monocytes have all been reported to support plasma cell survival by secretion of the survival factors APRIL and IL-6 (22–24). Furthermore, immunization of adult mice has been shown to activate eosinophils and trigger secretion of these survival factors (25). In neonatal mice, Mac1^+^F4/80^+^Gr-1^−^ bone marrow resident macrophages were reported to increase the survival of TT-specific plasmablasts following immunization (26). In neonatal mice most of the plasmablasts differentiated in germinal centers home efficiently to the bone marrow, but cannot differentiate into long-lived plasma cells and persist there due to lack of survival signals, such as APRIL. This is reflected in transient Ab responses in this age group (26).

Adjuvants are immune stimulating agents that can enhance both the induction and persistence of immune responses and may also affect the nature of responses. Alum was the only adjuvant included in licensed human infant vaccines until MF59 was included in the H1N1 pandemic influenza vaccine licensed in 2009 for vaccination from 6 months of age (27, 28). The adjuvant LT-K63 is a detoxified derivative of heat labile enterotoxin from *E. coli* that interacts with a variety of cells through binding of GM1 ganglioside (29). LT-K63 was originally developed as a mucosal adjuvant (30) and has passed a phase I clinical trial where it was administered mucosally with inactivated influenza vaccine, demonstrating protective Ab response and a good safety profile (31). In another study, two individuals experienced Bell's palsy, causing reconsideration of intranasal administration of this family of molecules (32). However, this molecule also elicits strong adjuvanticity when given parenterally (33, 34). We have reported that LT-K63 enhanced proliferation of splenocytes *in vitro* and secretion of IFN-γ, IL-4, and IL-10 by T cells following immunization of neonatal mice with pneumococcal conjugate vaccine, Pnc1-TT (35). LT-K63 also increased expression of activation- and co-stimulatory molecules CD86, CD40, and MHCII on B cells (35) and dendritic cells (36), which have been shown to be poorly expressed in neonates (37), that enables enhanced Ag-presenting capacity and increased interaction of these cells with T cells. Additionally, we have shown that immunization with Pnc1-TT with LT-K63 accelerated maturation of follicular dendritic cells, enhanced migration of marginal metallophilic macrophages into follicles and overcame limited induction of germinal center reaction in neonatal mice (38, 39). The increase in PPS-1- and TT-specific Ab-secreting cells (ASCs) in spleen and their long-term survival in bone marrow by LT-K63 (39) led to persistence of protective Abs in neonatal mice (33, 34).

The primary aim of this study was to assess through which factors and mechanisms LT-K63 exerts its effects on germinal center activation (38, 39) and sustained immune responses (33, 34, 39), focusing mainly on expression of TNF superfamily members. We used a pneumococcal conjugate vaccine that is immunogenic in early life (40), inducing T cell dependent responses, where induction of germinal center and their B cells play a critical role. First, we investigated whether acceleration of vaccine-induced humoral immune responses could be mediated through its effects on expression of BAFF-R and BCMA. Secondly, we evaluated for the first time in neonatal mice, which accessory cells of the neonatal plasma cell survival niche in the bone marrow secreted the plasma cell survival factor APRIL and if its secretion was affected by LT-K63.

## Materials and Methods

### Mice

We purchased adult (5–6 week old) NMRI mice from Taconic (Skensved, Denmark). After adapting for a week they were mated and kept in microisolator cages at the facility of ArcticLAS vivarium (Reykjavík, Iceland) under standardized conditions with regulated temperature, humidity, and daylight (38). They had free access to water and commercial food. The cages were checked daily for new births. Pups were kept with their mothers until weaning at 4 weeks of age.

### Vaccine and Adjuvants

The vaccine used in this study was a pneumococcal conjugate vaccine (Pnc1-TT) provided by Sanofi Pasteur (Marcy l'Etoile, France). It consisted of a pneumococcal polysaccharide (PPS) of serotype 1 (PPS-1) that was conjugated to tetanus toxoid (TT) (41). The LT-K63 adjuvant was produced as previously described (42, 43) and provided by Novartis Vaccines and Diagnostics (now GSK Vaccines, Siena, Italy).

### Immunizations

Neonatal (7 day old) mice (8 mice per group) were immunized once subcutaneously (s.c.) at scapular girdle with 0.5 μg of Pnc1-TT, with Pnc1-TT mixed with the adjuvant LT-K63 (5 μg per mouse) in 50 μl of saline or with saline as a control. Vaccine was mixed 1 h before immunizations.

### MACS Cell Separation

Bone marrow was collected 4, 8, 14, 21, and 56 days after priming. T cells were depleted from the bone marrow cells using anti-CD3ε-biotin and anti-biotin microbeads, mast cells, and basophils using anti-FcεRI-biotin and anti-biotin microbeads, B cells using CD45R (B220) microbeads and dendritic cells using CD11c (N418) microbeads. Microbeads were purchased from Miltenyi Biotec (Lund, Sweden), and we followed the manufacturer's protocol.

### Immunofluorescence Staining and Flow Cytometry

Spleens and bone marrow were collected 4, 8, 14, 21 and 56 days after immunization for flow cytometry analysis using the following protocol as described (35). We prepared single-cell suspensions from spleen and bone marrow (for assessing accessory cells, the bone marrow cells were depleted of several cell types as described in section MACS cell separation). Cells were washed and incubated (30 min on ice) in PBS with 0.5% BSA (Sigma) with 4 mmol/L EDTA (Sigma) with fluorochrome-labeled antibodies to B220, CD21, CD23, BAFF-R, CD138, F4/80, CD11b, Gr-1, Siglec-F, and APRIL (all from BD Biosciences) and BCMA (R&D Systems). Fc block (BD Biosciences), rat serum and mouse serum (2,7% each) was added to the staining mix to minimize unspecific binding. The stained cells were analyzed using FACSCalibur (BD Biosciences) where recorded events were 100,000 cells and Navios cytometer (Beckman Coulter, Brea, CA, USA) where recorded events were 400,000, and the generated data were analyzed by Kaluza® analysis software (version 1.3 from Beckman Coulter) where dead cells and doublets were excluded prior to analysis.

### Immunofluorescence Staining of Tissue Sections

Spleens collected 8 and 14 days after immunization were frozen using Tissue-Tek OCT (Sakura, Zouterwoude, the Netherlands). They were cut into cryosections (7 μm) at 2 levels, the first starting 1,750 μm into the tissue and the second level was separated from the first level by 210 μm. The sections were fixed for 10 min in acetone, and stored at −70°C. For peanut agglutinin (PNA) and BAFF/APRIL staining, two adjacent sections from each level of the spleen from each mouse were stained. The prior section from each level was stained with rabbit-anti-mouse polyclonal BAFF Ab and the latter with rabbit-anti-mouse polyclonal APRIL Ab (Invitrogen, Eugene, OR, USA), incubated for 30 min at room temperature as described (39). The sections were washed with PBS twice for 5 min before incubation with secondary Ab, Alexa Fluor™ 488-labeled F(ab′)2 fragment of goat anti-rabbit IgG (H+L) (Invitrogen) for 30 min with blocking solution (rabbit serum, goat serum, rat serum, and Fc block, 5% each) at room temperature and then washed as before. The sections were then stained with biotinylated PNA (Vector Laboratories, Burlingame, CA) for 30 min at room temperature and washed as before, followed by a 30 min incubation with APC labeled Streptavidin (BD Biosciences). For IgM and PNA staining (Supplementary Figure 6) one section from each level for each mouse was stained using fluorescent labeled IgM-FITC (BD Pharmingen) to visualize follicles, and PNA-bio (Vector Laboratories, Burlingame, CA) to label dark-zone B cells identifying active germinal center reaction. The sections were washed as described above and then incubated with APC labeled Streptavidin (BD Biosciences) for 30 min before washing. Positive cells in each section were counted by two individuals, blinded regarding their identity, and photograhps were taken in a microscope AxioImager (Zeiss) using digital camera (AXIOCAM; Zeiss) and analyzed as before (39) by AxioVision Software (Birkerod, Denmark).

### ELISA

Blood samples were collected from the vein of tail 14, 21, and 56 days after one immunization at 7 days of age and serum isolated for storage at −20°C until use. PPS-1- and TT-specific Abs (IgG) were measured in sera by ELISA, as described (44). In short, we coated microtiter plates (MaxiSorp; Nunc AS, Roskilde, Denmark) with 5 μg PPS-1 (American Type Culture Collection, Rockville, MD) in PBS for 5 h at 37°C or with 5 μg TT (Statens Serum Institute, Copenhagen, Denmark) per ml of 0.10 M carbonate buffer (pH 9.6) overnight at 4°C. The plates were washed and blocked with PBS-Tween 20 containing 1% BSA (Sigma). Serum samples and standard were neutralized with cell wall polysaccharide (Statens Serum Institute) for 30 min before they were serially diluted in the plate and incubated for 2 h at room temperature. Plates were washed and then incubated with HRP-conjugated goat anti-mouse Ab (Southern Biotechnology Associates, Birmingham, AL). Plates were washed again and 3,3′,5,5′-tetramethylbenzidine-substrate (Kirkegaard & Perry Laboratories, Gaithersburg, MD) was used for development and the reaction stopped with 0.18 M H_2_SO_4_. The color density at 450 nm was read in Titertek Multiscan Plus MK II spectrophotometer (ICN Flow Laboratories, Irvine, UK). We calculated the results from a standard curve and expressed them as mean log of ELISA units (EU)/ml as described (44). The standard for both PPS- and TT-specific Abs was obtained by isolating sera from adult mice hyper-immunized with the conjugate vaccine. The titers of the standards (EU/milliliters) corresponded to the inverse of the serum dilution giving an optical density of 1.0.

### ELISPOT

We enumerated ASCs specific for PPS-1 and TT in spleen 14 and 21 days after immunization and in bone marrow 56 days after immunization using ELISPOT, as described (39, 45). We coated MultiScreen High protein binding immobilon-P membrane plates (Millipore Corporation, Bedford, MA) overnight at 37°C with 20 μg PPS-1 per ml or with 10 μg TT per ml. Plates were then blocked with complete RPMI 1640 (Life Technologies BRL, Life Technologies, Paisley, U.K.) for 1 h, spleen, or bone marrow cells (10^8^ cells per ml) were serially diluted in complete RPMI 1640 (39, 45) and incubated at 37°C for 5 h. The plates were then washed and incubated at 4°C overnight with ALP-conjugated goat anti-mouse IgG (Southern Biotechnology Associates) and the reaction was developed using AP development substrate kit (Bio-Rad Labs, Hercules, CA) containing 5-bromo-4-chloro-3-indolylphosphate (BICIP) and nitroblue tetrazolium (NBT). We used ELISPOT reader ImmunoSpot® S6 ULTIMATE to count the spots and ImmunoSpot® SOFTWARE (Cellular Technology Limited (CTL) Europe, Bonn, Germany) for analysis as described (38).

### Statistical Analysis

For comparison of groups we used Mann-Whitney U test, applying a significance threshold of *p* < 0.05. Graphpad Prism 8 (GraphPad Software, La Jolla, CA) was used to perform all statistical analyses.

## Results

### B Cell Subsets in Spleen and Their Expression of BAFF-R and BCMA in Early Life

**Figure 1:**
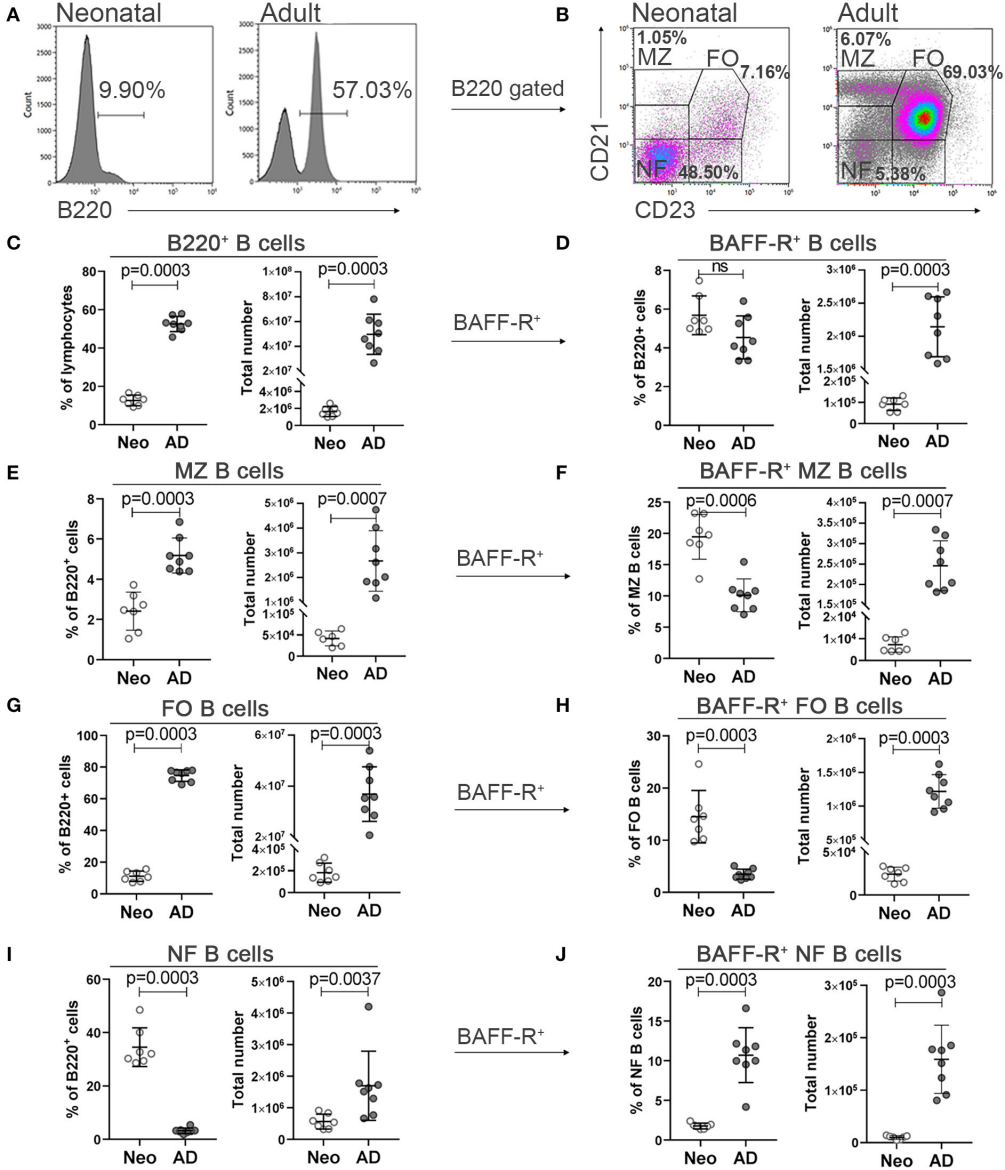
Neonatal and adult B cell subsets in spleen and their expression of BAFF-R. Representative histograms and gating of B220^+^ cells **(A)** representative dot plots and gating strategies for the following B220^+^ cell subsets; marginal zone (MZ; CD21^high^CD23^−/low^), follicular (FO; CD21^+^CD23^+^) and newly formed (NF; CD21^−^ CD23^−^) B cells **(B)** in neonatal (Neo; 7 days old) and adult mice (AD; 8–10 weeks). Frequency, total number, proportional BAFF-R expression and total number of B220^+^BAFF-R^+^ cells **(C,D)**, marginal zone B cells **(E,F)**, follicular B cells **(G,H)** and newly formed B cells **(I,J)** assessed by flow cytometry. For statistical evaluation Mann–Whitney *U*-test was used.

We first assessed the developmental status of different subsets of B cells in neonatal and adult mice and their expression of BAFF-R in spleen. Following gating of B220^+^ B cells, newly formed B cells were defined as CD21^−^CD23^−^, follicular B cells as CD21^+^CD23^+^ and marginal zone B cells as CD21^high^CD23^−/low^ (Figures 1A,B). Compared to adult mice, neonatal mice had a higher frequency of newly formed B cells (Figure 1I), but lower frequency of B220^+^ B cells (Figure 1C), marginal zone (Figure 1E) and follicular B cells (Figure 1G). However, when assessing total number of cells, neonatal mice had fewer cells in all subsets (Figures 1C,E,G,I). There was no difference in the frequency of BAFF-R^+^ cells among B220^+^ B cells between neonatal and adult mice (Figure 1D). Newly formed B cells of neonatal mice had diminished expression of BAFF-R (Figure 1J). On the contrary, a higher proportion of marginal zone and follicular B cells was BAFF-R^+^ in neonatal than adult mice (Figures 1F,H).

**Figure 2:**
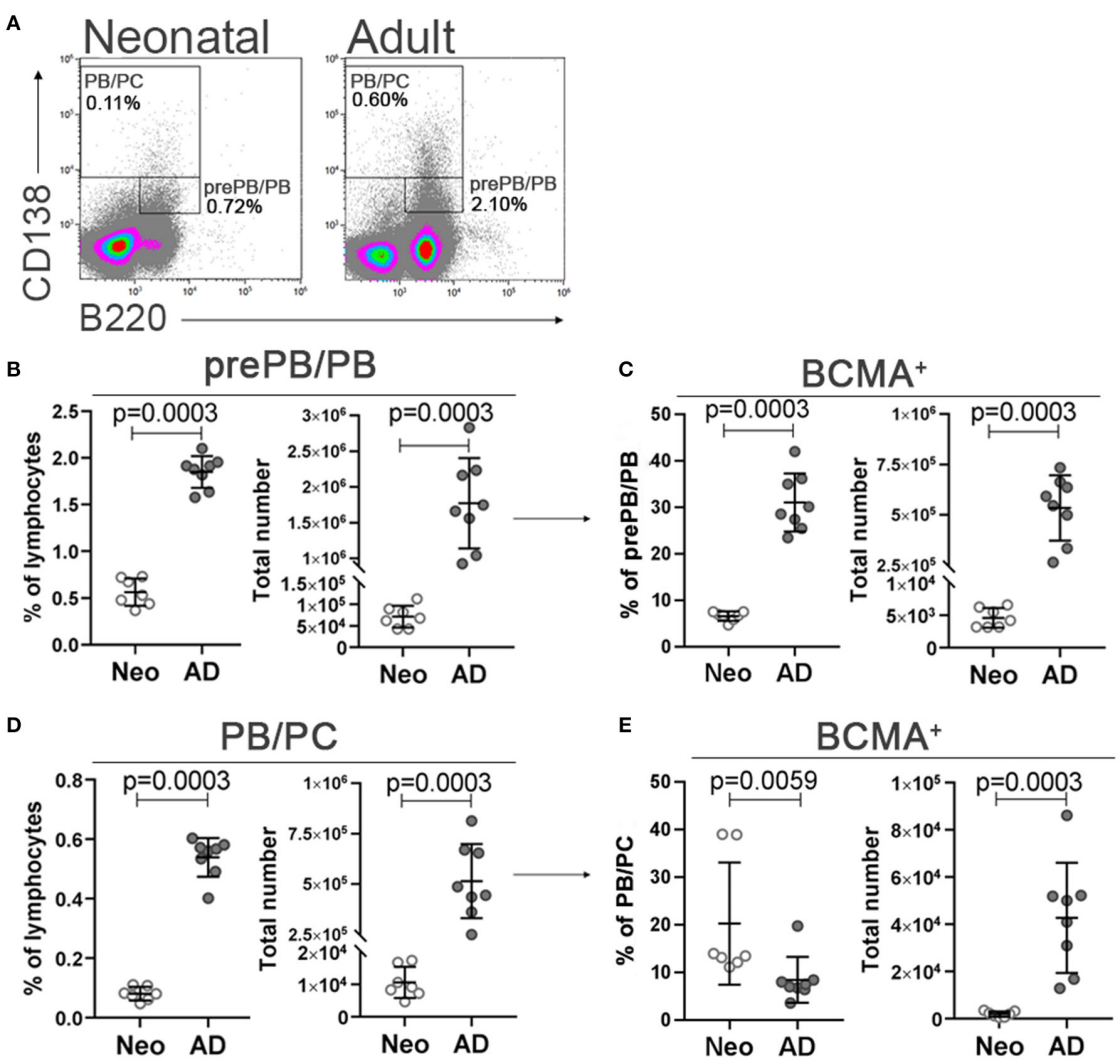
Neonatal and adult plasmablasts and plasma cells and their expression of BCMA in spleen. Representative dot plots and gating strategy of prePB/PB (B220^+^CD138^int^ cells, containing pre-plasmablasts, and plasmablasts) and PB/PC (B220^+/−^CD138^high^, containing plasmablasts, and plasma cells) in neonatal (7 days old) and adult (8–10 weeks) mice **(A)**. Frequency and total number of prePB/PB **(B)** and their BCMA expression **(C)** and frequency and total number PB/PC **(D)** and their BCMA expression **(E)** in neonatal (Neo) and adult (AD) mice. For statistical evaluation Mann–Whitney *U*-test was used.

We also assessed the developmental status of plasmablasts and plasma cells and their expression of BCMA in spleen of neonatal and adult mice. We observed lower expression of CD138 in neonates with decreased B220^+/−^CD138^high^ /B220^+^CD138^int^ ratio (0.15 ± 0.04 in neonatal mice and 0.29 ± 0.03 in adult mice, data not shown), indicating a possible limitation in early life CD138 expression. Therefore, we assessed B220^+^CD138^int^ cells, reported to include pre-plasmablasts and plasmablasts in neonatal and adult mice (26, 46), from now on referred to as prePB/PB, and B220^+/−^CD138^high^ cells recently shown to contain both plasmablasts and plasma cells in adult mice (46, 47), from now on referred to as PB/PC (Figure 2A). Adult mice had higher frequencies and total number of both subsets assessed; prePB/PB and PB/PC (Figures 2B,D). When BCMA expression of the subsets was assessed, a lower proportion of prePB/PB expressed BCMA whereas a higher proportion of PB/PC was BCMA^+^ in neonatal than adult mice. However, neonatal mice had lower numbers of BCMA^+^ prePB/PB and PB/PC cells (Figures 2C,E).

### LT-K63 Enhances BAFF-R Expression in B Cell Subsets in Spleen

**Figure 3:**
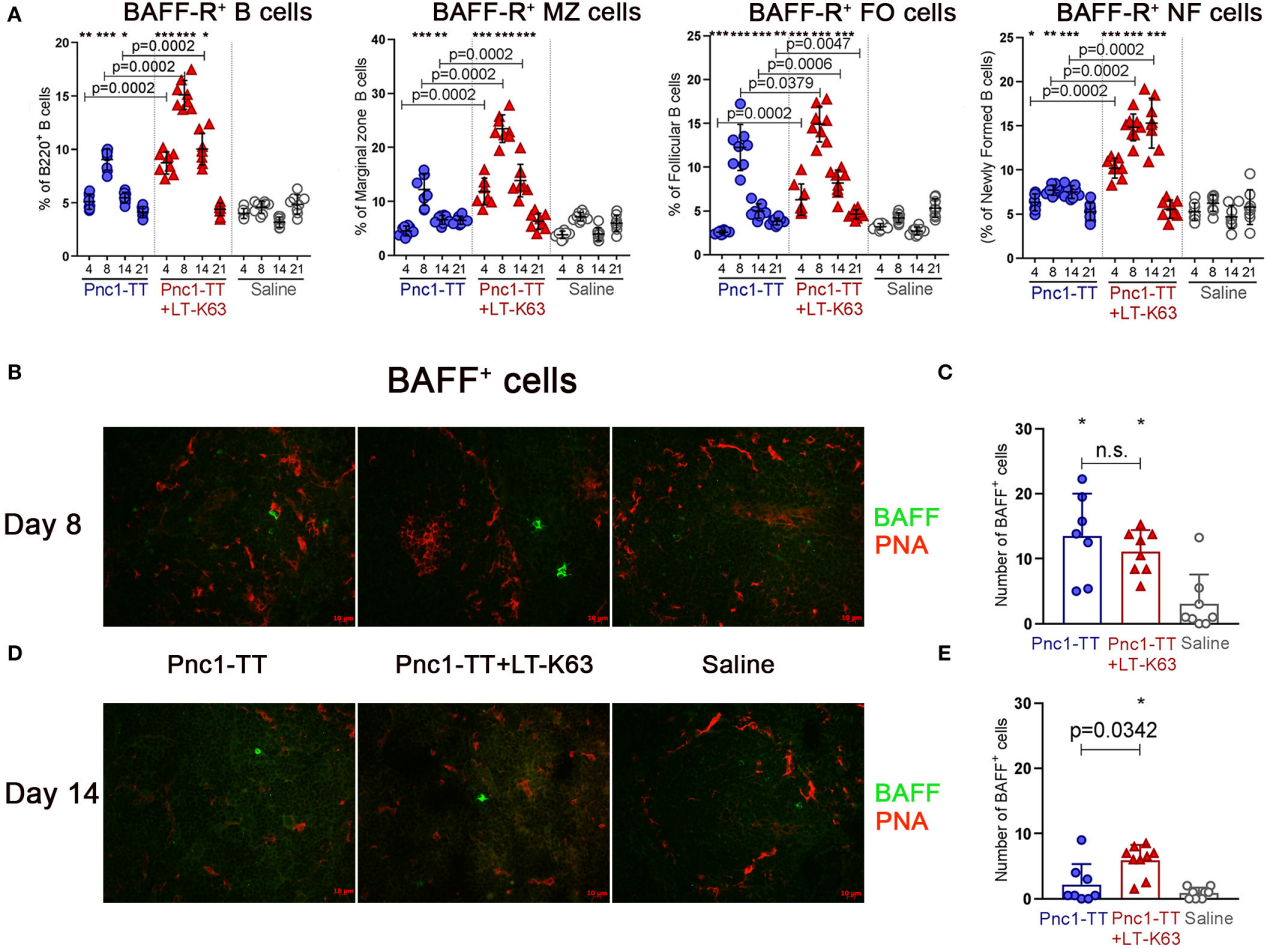
LT-K63 increases proportional expression of BAFF-R in B220^+^ B cells, marginal zone, follicular, and newly formed B cells and BAFF^+^ cells in spleen. Proportion of BAFF-R expression on B220^+^, marginal zone (CD21^high^CD23^−/low^), follicular (CD21^+^CD23^+^) and newly formed (CD21^−^ CD23^−^) B cells at 4, 8, 14, and 21 days following neonatal immunization with Pnc1-TT (blue), Pnc1-TT+LT-K63 (red), or saline (white) as a control, assessed by flow cytometry **(A)**. Representative immunofluorescense staining pattern for BAFF^+^ cells of each group in spleen 8 **(B)** and 14 days **(D)** after immunization. Co-staining of peanut agglutinin (PNA, red) was included to demonstrate localization of positive cells. Original magnification ×40. Scale bar, 10 μm. BAFF^+^ cells were counted per spleen section by two individuals (blinded regarding the identity of the sections) for each spleen at two levels and results are shown in spleen 8 **(C)** and 14 days **(E)** after immunization with Pnc1-TT (blue), Pnc1-TT+LT-K63 (red), or saline (white). Each symbol represents one mouse and results are shown as means±SD in 8 mice per group per time point. For statistical evaluation Mann–Whitney *U*-test was used. *P*-vaules are shown for the comparison of Pnc1-TT group to Pnc1-TT+LT-K63, stars represent comparisons of Pnc1-TT or Pnc1-TT+LT-K63 groups to saline group. *p ≤ 0.05, **p ≤ 0.01, ***p ≤ 0.001.

We then assessed the effect of LT-K63 on BAFF-R expression in the different B cell subsets. Neonatal mice were immunized s.c. with 0.5 μg of Pnc1-TT alone or mixed with the adjuvant LT-K63 or with saline only as a control. The adjuvant LT-K63 enhanced BAFF-R expression, as higher proportion of B220^+^ B cells and all subsets assessed; marginal zone, follicular, and newly formed B cells, was BAFF-R^+^ in mice immunized with Pnc1-TT+LT-K63 than Pnc1-TT alone 4, 8, and 14 days after immunization (Figure 3A). Immunization with Pnc1-TT alone also enhanced BAFF-R expression among the B cell subsets compared to saline, but not to the same extent as when the adjuvant was included in the vaccine formulation.

Several cell types, including B cells (48), myeloid cells [reviewed in (49)] and follicular dendritic cells (50) express the TNF-R ligand BAFF. Spleen sections obtained 8 and 14 days after immunization were stained for BAFF and with peanut agglutinin (PNA), that is a good marker for active germinal center and also stains endothelium like the marginal sinus. Immunization of neonatal mice with Pnc1-TT either with or without LT-K63 enhanced BAFF expression in spleen 8 days after immunization compared to mice receiving saline (Figures 3B,C). The expression was better maintained in mice that received the adjuvant, as they had significantly higher number of BAFF^+^ cells 14 days after immunization than mice that received the vaccine alone (Figures 3D,E). BAFF^+^ cells were commonly located in the follicles.

Taken together, these results show that the adjuvant LT-K63, which restores limited germinal center induction in neonatal mice (38, 39), also contributes to an increased activation of neonatal B cells, partly through enhanced expression of BAFF-R on follicular, marginal zone, and newly formed B cells and more prolonged expression of its ligand, BAFF, in the spleen.

### LT-K63 Increases Frequency and BCMA Expression of Splenic Plasmablasts and Plasma Cells

To explore whether the increased expression of BAFF and BAFF-R observed after immunization with LT-K63-adjuvanted Pnc1-TT led to increased differentiation of B cells to plasmablasts, we assessed the frequency and BCMA expression of plasmablasts and plasma cells in spleens 4, 8, 14, and 21 days after immunization. The analyzed populations were defined as above; prePB/PB (B220^+^CD138^int^ cells containing pre-plasmablasts and plasmablasts) and PB/PC (B220^+/−^CD138^high^ cells containing plasmablasts and plasma cells).

**Figure 4:**
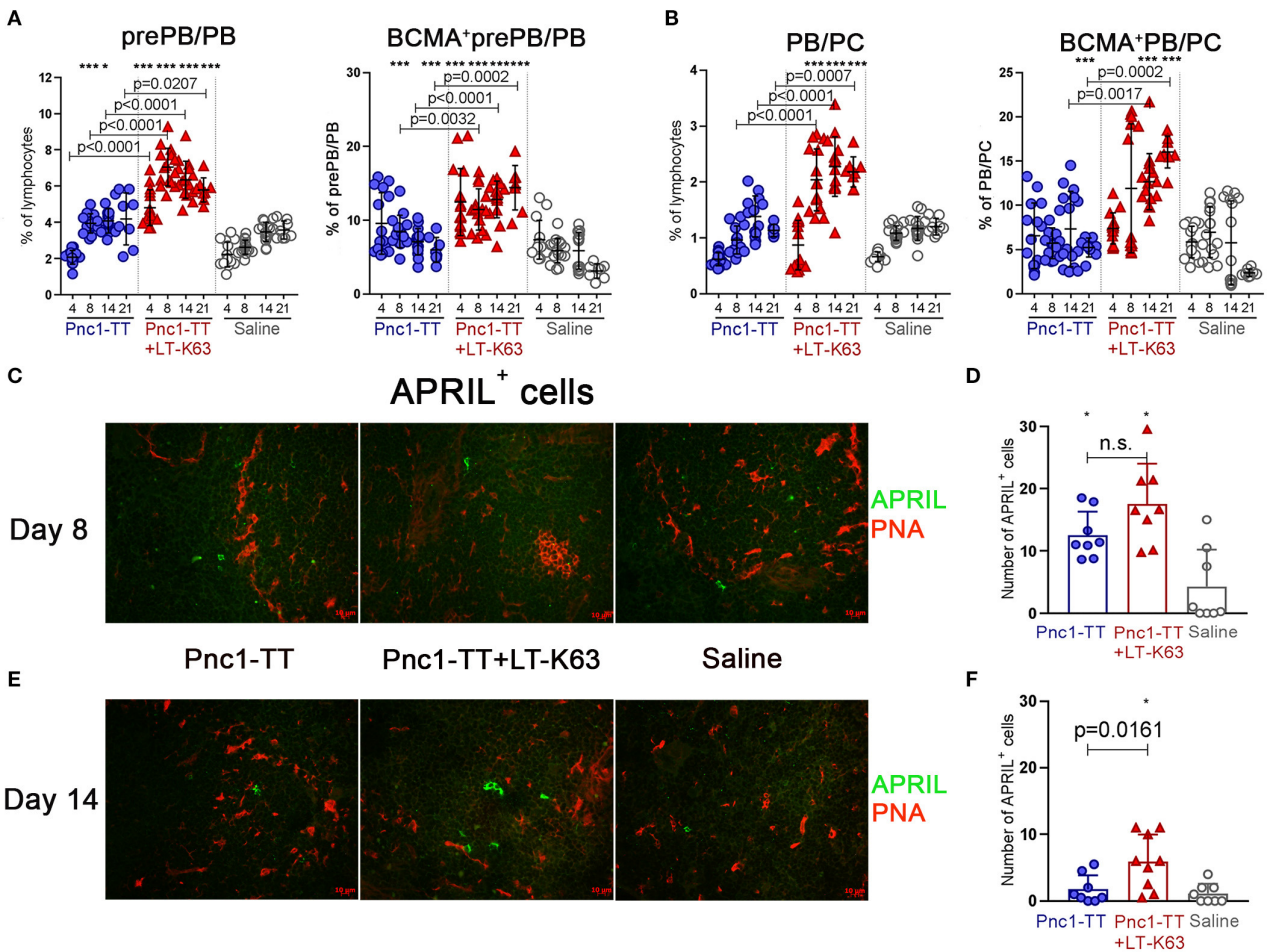
LT-K63 increases frequency of plasmablasts, plasma cells, their expression of BCMA and prolongs APRIL secretion in spleen. Frequency of prePB/PB (B220^+^CD138^int^ cells, containing pre-plasmablasts, and plasmablasts) and their BCMA expression **(A)**, PB/PC (B220^+/−^CD138^high^, containing plasmablasts and plasma cells) and their BCMA expression **(B)** in spleen 4, 8, 14, and 21 days after neonatal immunization with Pnc1-TT (blue), Pnc1-TT+LT-K63 (red), or saline (white) as a control, assessed by flow cytometry. Each symbol represents one mouse and results are shown as means ± SD. Data is pooled from two independent experiments for days 4, 8, and 14 (*n* = 7–8 for each group per time point each experiment) but data for day 21 represents one experiment (*n* = 8 per group per time point). Representative immunofluorescense staining pattern for APRIL^+^ cells (green) in spleen 8 **(C)** and 14 days **(E)** after immunization. Co-staining of peanut agglutinin (PNA, red) was included to demonstrate localization of positive cells. Original magnification ×40. Scale bar, 10 μm. APRIL^+^ cells per spleen section were counted by two individuals (blinded regarding the identity of the sections) for each spleen at two levels and results are shown as means ± SD in 8 mice per group in spleen 8 **(D)** and 14 days **(F)** after immunization. For statistical evaluation Mann–Whitney *U*-test was used. *P*-vaules are shown for the comparison of Pnc1-TT group to Pnc1-TT+LT-K63, stars represent comparisons of Pnc1-TT or Pnc1-TT+LT-K63 groups to saline group. *p ≤ 0.05, ***p ≤ 0.001.

The adjuvant LT-K63 enhanced frequency and total number of both prePB/PB and PB/PC compared to Pnc1-TT alone (Figures 4A,B and Supplementary Figures 2A,B). It is worth noting that a higher proportion of prePB/PB expressed BCMA at days 8, 14, and 21 after immunization with Pnc1-TT+LT-K63 than Pnc1-TT alone (Figure 4A and Supplementary Figure 2E). The same was observed for BCMA expression of PB/PC 14 and 21 days after immunization (Figure 4B and Supplementary Figure 2F).

Since the BCMA receptor binds the cytokine APRIL, that is an essential survival factor for plasma cells (26), we performed immunofluorescence staining of APRIL together with PNA in spleen sections. Although no difference was found in the number of APRIL^+^ cells 8 days after immunization (Figures 4C,D), at day 14 mice immunized with Pnc1-TT+LT-K63 had a significantly higher number of APRIL^+^ cells than mice immunized with Pnc1-TT alone (Figures 4E,F). APRIL^+^ cells were preferentially located in extrafollicular focis.

**Figure 5:**
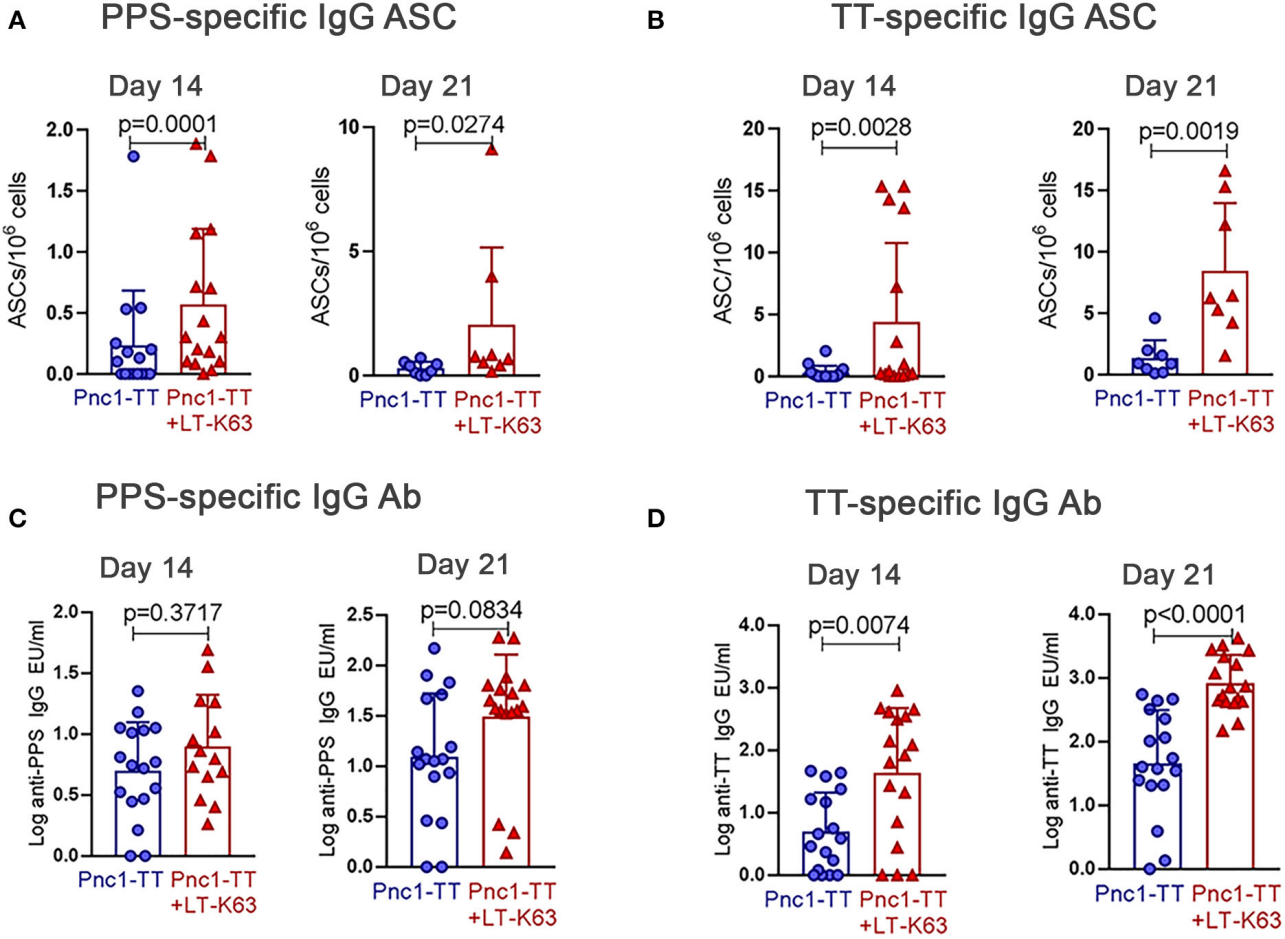
LT-K63 increases vaccine-specific antibody-secreting cells and antibodies. PPS-specific **(A,C)** and TT-specific **(B,D)** antibody-secreting cells in spleen **(A,B)** and antibodies in serum **(C,D)** 14 and 21 days after neonatal immunization with Pnc1-TT (blue) or Pnc1-TT +LT-K63 (red). Each symbol represents one mouse and results are shown as means ± SD. Data is pooled from two independent experiments (*n* = 7–8 for each group per time point each experiment) but data for ASC at day 21 represents one experiment (*n* = 8 per group per time point). For statistical evaluation Mann–Whitney *U*-test was used.

In agreement with what we had previously shown (39), mice immunized with LT-K63+Pnc1-TT had higher numbers of both PPS- and TT-specific ASCs in spleen than mice that only received Pnc1-TT, 14 and 21 days after immunization (Figures 5A,B). Those mice also had significantly higher levels of TT-specific serum IgG Abs at both time points but there was not a significant difference in levels of PPS-specific IgG Abs (Figures 5C,D). Therefore, increased expression of BAFF-R on B cell subsets leads to a higher proportion of activated B cells that in turn can lead to differentiation of more plasmablasts and fully mature ASC that are possibly better able to survive due to increased expression of BCMA and prolonged secretion of its ligand, APRIL.

### LT-K63 Increases BCMA Expression on Plasmablasts and Plasma Cells in Bone Marrow

**Figure 6:**
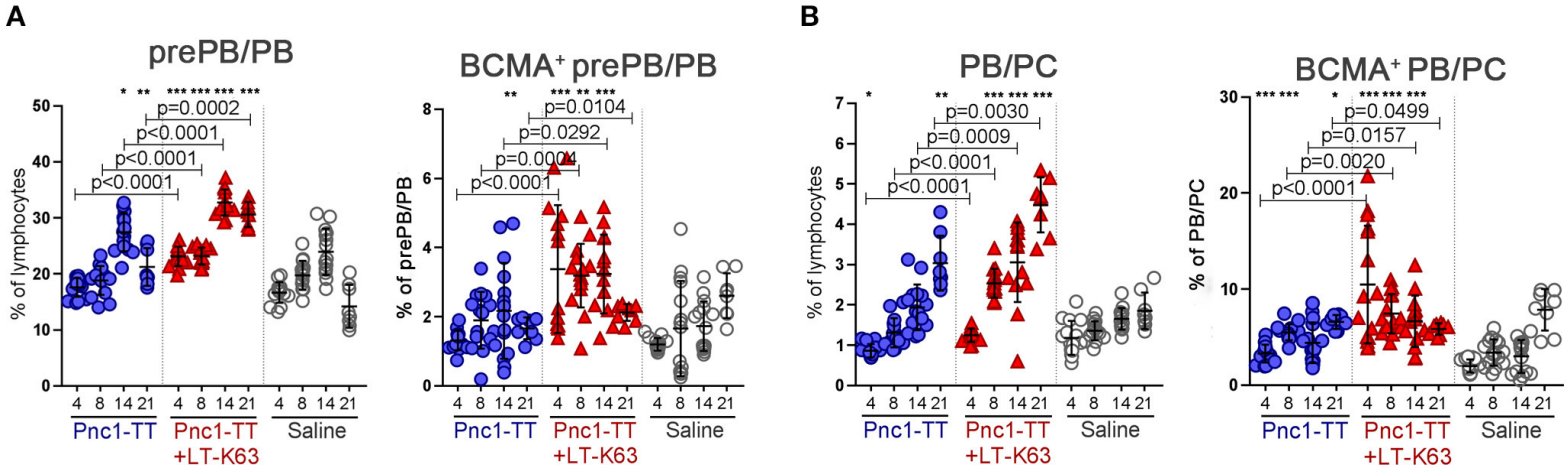
LT-K63 increases frequency of plasmablasts and plasma cells and their expression of BCMA in bone marrow. Frequency of prePB/PB (B220^+^CD138^int^ cells, containing pre-plasmablasts, and plasmablasts) and their BCMA expression **(A)**, PB/PC (B220^+/−^CD138^high^, containing plasmablasts, and plasma cells) and their BCMA expression **(B)** in bone marrow 4, 8, 14, and 21 days after neonatal immunization with Pnc1-TT (blue), Pnc1-TT+LT-K63 (red), or saline (white) as a control, assessed by flow cytometry. Each symbol represents one mouse and results are shown as means ± SD. Data is pooled from two independent experiments for days 4, 8, and 14 (*n* = 7–8 for each group per time point each experiment) but data for day 21 represents one experiment (*n* = 8 per group per time point). For statistical evaluation Mann–Whitney *U*-test was used. *P*-vaules are shown for the comparison of Pnc1-TT group to Pnc1-TT+LT-K63, stars represent comparisons of Pnc1-TT or Pnc1-TT+LT-K63 groups to saline group. *p ≤ 0.05, **p ≤ 0.01, ***p ≤ 0.001.

Plasma cells formed in germinal centers in secondary lymphoid tissues home to the bone marrow and can differentiate there into long-lived plasma cells and persist for a long time in specialized survival niches (51). We next examined whether the LT-K63-increased BCMA expression on plasmablasts and plasma cells in spleen led to their enhanced survival in the bone marrow. Mice immunized with Pnc1-TT+LT-K63 had higher frequencies of prePB/PB (B220^+^CD138^int^) and PB/PC (B220^+/−^CD138^high^) cells in bone marrow at all time point assessed than mice immunized with Pnc1-TT only (Figures 6A,B and Supplementary Figures 3A,B). Moreover, LT-K63 enhanced BCMA expression of these cells, as a higher portion of prePB/PB and PB/PC was BCMA^+^ in the Pnc1-TT+LT-K63 group than in Pnc1-TT group (Figures 6A,B and Supplementary Figures 3C–F). We have previously shown that LT-K63 increases the survival of ASC in bone marrow (39). Herein, we show that this can at least partly be due to increased BCMA expression of plasmablasts and plasma cells in spleen and bone marrow.

### LT-K63 Increases Frequency of Eosinophils, Macrophages, and Megakaryocytes and Their Secretion of APRIL in Bone Marrow

To further investigate mechanisms mediating prolonged survival of plasma cells in the bone marrow, we assessed, for the first time in neonatal mice, the accessory cells in the plasma cell survival niche, eosinophils (Gr-1^int^F4/80^+^CD11b^+^Siglec-F^+^SSC^high^), macrophages (Gr-1^int^F4/80^+^CD11b^+^Siglec-F^int^SSC^int^), and megakaryocytes (CD41^+^FSC^hi^), and their expression of the plasma cell survival factor APRIL (Supplementary Figure 4).

**Figure 7:**
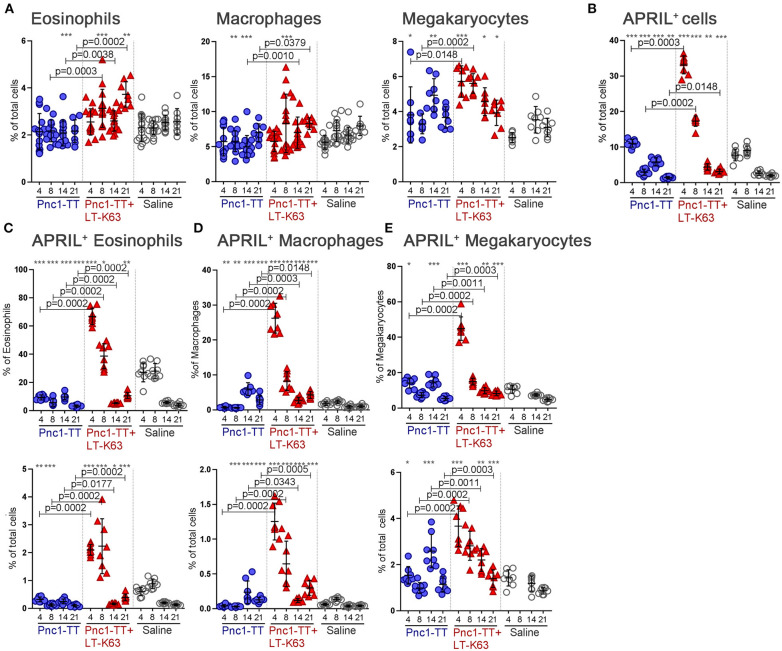
LT-K63 increases frequency of eosinophils, macrophages and megakaryocytes and their expression of APRIL in bone marrow. Frequency of eosinophils (Gr-1^int^F4/80^+^CD11b^+^Siglec-F^+^SSC^high^), macrophages (Gr-1^int^F4/80^+^CD11b^+^Siglec-F^int^SSC^int^) and megakaryocytes (CD41^+^FSC^hi^) **(A)**, APRIL^+^ cells **(B)**, proportional APRIL expression and frequency of APRIL^+^ eosinophils **(C)**, APRIL^+^ macrophages **(D)** and APRIL^+^ megakaryocytes **(E)** in bone marrow 4, 8, 14, and 21 days after neonatal immunization with Pnc1-TT (blue), Pnc1-TT+LT-K63 (red), or saline (white), assessed by flow cytometry. Bone marrow suspensions were depleted of B cells, T cells, dendritic cells, mast cells, and basophils before flow cytometry analysis. Each symbol represents one mouse and results are shown as means ± SD for 8 mice per group per time point except for day 4, 8, and 14 in **(A)** were two independent experiments are pooled for frequency of eosinophils and macrophages (7–8 mice per group per time point per experiment). For statistical evaluation Mann–Whitney *U*-test was used. *P*-vaules are shown for the comparison of Pnc1-TT group to Pnc1-TT+LT-K63, stars represent comparisons of Pnc1-TT, or Pnc1-TT+LT-K63 groups to saline group. *p ≤ 0.05, **p ≤ 0.01, ***p ≤ 0.001. Gating strategy for eosinophils and macrophages is displayed in Supplementary Figure 4.

Overall, the adjuvant LT-K63 enhanced the frequency of eosinophils, macrophages, and megakaryocytes in bone marrow, yet with different kinetics (Figure 7A). It also induced a major increase in the frequency of APRIL^+^ cells early after immunization. The frequency of APRIL^+^ cells was highest 4 days after immunization when over 30% of all analyzed cells (after depletion) were APRIL^+^ in the Pnc1-TT+LT-K63 immunized group. By day 8 it had dropped to 15–20% in this group and by day 14 it was around 5%. APRIL^+^ cells reached a maximum of 10% in the Pnc1-TT group 4 days after immunization (Figure 7B). Furthermore, eosinophils, macrophages, and megakaryocytes were more frequently APRIL^+^ in mice immunized with Pnc1-TT+LT-K63 than Pnc1-TT 4, 8, and 21 days after immunization (Figures 7C–E).

Taken together, we can reasonably conclude that that the increased survival of ASCs induced by LT-K63 is at least partly due to increased early expression of APRIL by these accessory cells in the bone marrow survival niches.

### LT-K63 Induces Persistent Humoral Immune Responses

**Figure 8:**
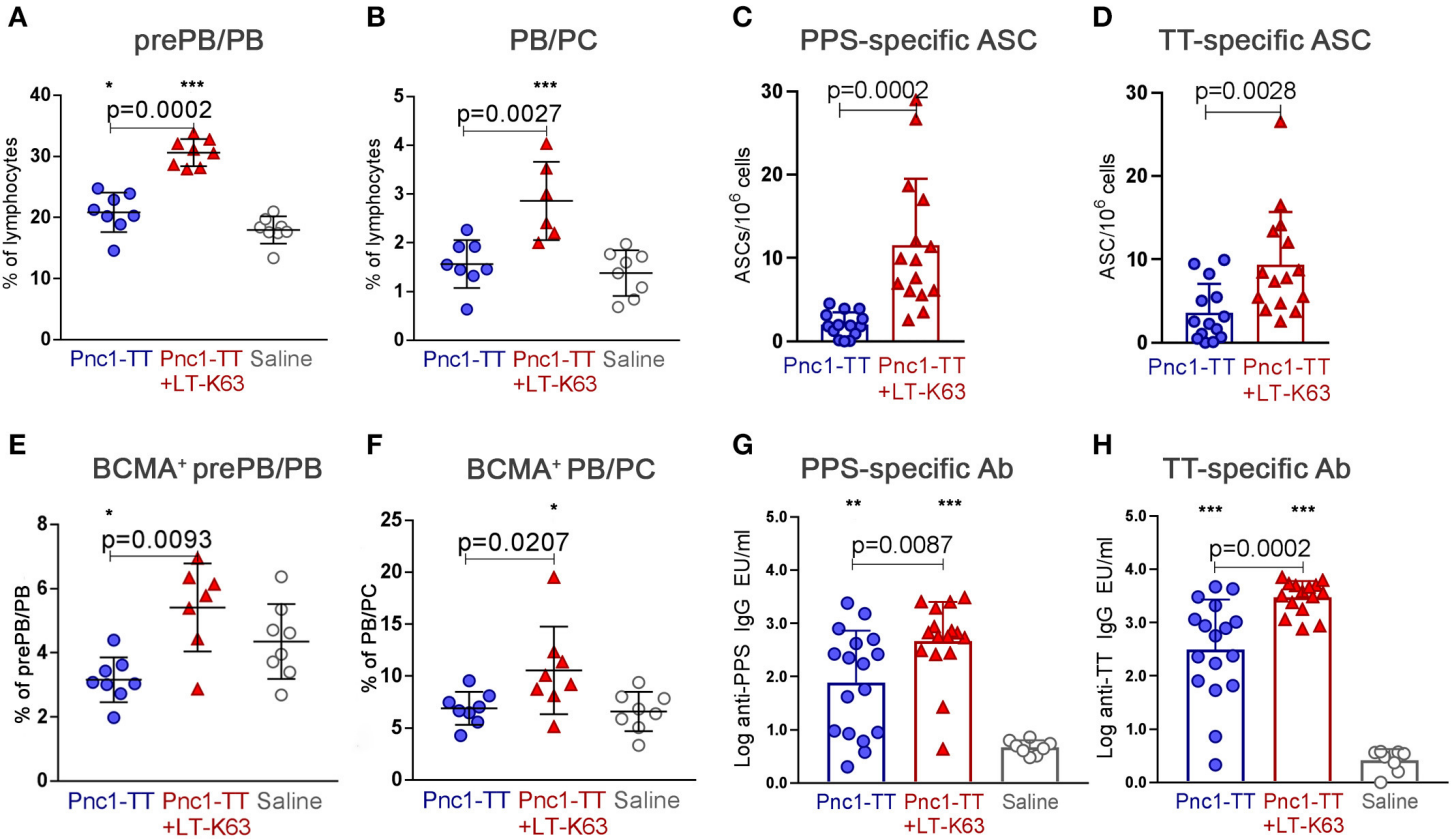
LT-K63 increases BCMA expression and persistency of vaccine-specific antibody-secreting cells in bone marrow and vaccine-specific serum antibodies. Frequency of prePB/PB (B220^+^CD138^int^ cells, containing pre-plasmablasts, and plasmablasts) **(A)** and their BCMA expression **(E)**, PB/PC (B220^+/−^CD138^high^, containing plasmablasts and plasma cells) **(B)** and their BCMA expression **(F)** in bone marrow 8 weeks after neonatal immunization with Pnc1-TT (blue), Pnc1-TT+LT-K63 (red), or saline (white) as a control, assessed by flow cytometry, PPS-specific antibody-secreting cells in bone marrow **(C)** and TT-specific antibody-secreting cells in bone marrow **(D)** enumerated by ELISPOT and PPS-specific serum antibodies **(G)** and TT-specific serum antibodies **(H)** 8 weeks after immunization of neonatal mice with Pnc1-TT (blue), Pnc1-TT+LT-K63 (red), or saline (white). Each symbol represents one mouse and results are shown as means ± SD for 6–8 mice per group from one experiment in **(A,B,E,F)**. Data in **(C,D,G,H)** is pooled from two independent experiments (*n* = 8 for each group each experiment). For statistical evaluation Mann–Whitney *U*-test was used. *P*-values are shown for the comparison of Pnc1-TT group to Pnc1-TT+LT-K63, stars represent comparisons of Pnc1-TT or Pnc1-TT+LT-K63 groups to saline group. *p ≤ 0.05, **p ≤ 0.01, ***p ≤ 0.001.

Lastly, we examined if the increased expression of TNF receptors and their ligands demonstrated in this study associated with persistent ASC in bone marrow and Ab responses 8 weeks after immunization. Mice immunized as neonates with Pnc1-TT+LT-K63 8 weeks earlier still had higher frequency of prePB/PB (B220^+^CD138^int^) (Figure 8A) and PB/PC (B220^+/−^CD138^high^) (Figure 8B and Supplementary Figures 5A,D) in bone marrow than mice immunized with Pnc1-TT only. A higher fraction of prePB/PB and PB/PC from those mice were BCMA^+^ at this time point (Figures 8E,F and Supplementary Figures 5B,C,E,F). Mice immunized with Pnc1-TT+LT-K63 also had higher numbers of PPS- and TT-specific ASCs in bone marrow (Figures 8C,D) and increased levels of PPS- and TT-specific serum Abs than mice who received only Pnc1-TT, 8 weeks after immunization (Figures 8G,H). Thus, LT-K63-enhanced expression of TNF receptors BAFF-R and BCMA, as well as their ligands BAFF and APRIL, associated with persistent humoral immune responses after only one immunization.

## Discussion

The data presented here shed light on molecular mechanisms of adjuvanticity of LT-K63 following parenteral immunization, how enhanced expression of BAFF-R and BCMA on B cells and maintained secretion of BAFF and APRIL precedes the induction of germinal centers (39) and translates into increased survival of ASC and persistent Ab responses in neonatal mice. We have herein defined new markers of adjuvanticity that may be used in future research on other adjuvants for potential use in early life.

As previously reported (12), we found that neonatal mice had significantly lower proportion of total B220^+^ B cells among lymphocytes than adult mice (Figure 1C) explaining low humoral responses in this age group (52). Additionally, the composition of different subsets; the follicular, marginal zone, and newly formed B cells, within the B220^+^ B cell pool, differs greatly between neonatal and adult mice (Figures 1E,G,I), with higher frequencies of newly formed B cells and less follicular and marginal zone B cells in neonatal mice.

BAFF-R is important for the developmental maturation of B cells and modulates B cell survival and differentiation during activation (53, 54). Lower proportion of newly formed B cells, but higher proportions of B220^+^ B cells, marginal zone, and follicular B cells were BAFF-R^+^ in neonatal than adult mice (Figures 1D,F,H,J). The low number of follicular and marginal zone B cells observed in neonates could be explained by diminished expression of BAFF-R in newly formed B cells, as BAFF is one of the essential survival factors needed for their maturation and differentiation into follicular or marginal zone B cells (55, 56).

Immunization with Pnc1-TT increased BAFF-R (Figure 3A) and BAFF expression (Figures 3C,E) early after immunization. However, when LT-K63 was included the increase was more pronounced and better maintained coinciding with the peak of germinal center reaction 14 days after immunization (Supplementary Figure 6) (5, 39). BAFF is required for efficient selection of high affinity germinal center B cells (57) which can explain how LT-K63 is able to overcome limited germinal center induction in neontal mice, as we have previously shown (38, 39). Poorly maintained BAFF in spleen and low BAFF-R expression may contribute to transient responses induced by Pnc1-TT and Pnc1-TT+alum (38). In contrast we have recently shown that the adjuvants mmCT, MF59, and IC31 all enhanced germinal center induction and induced persistent immune responses after only one immunization with Pnc1-TT (38). Additionally, the adjuvant CAF01 has been shown to induce germinal centers in neonatal mice (58). It is unknown whether the adjuvanticity of these adjuvants is mediated through the same mechanisms as those of LT-K63, since their effects on TNF receptors and their ligands in neonatal mice have not been reported.

Expression of CD138 was decreased in neonatal compared to adult mice, thus CD138^int^ cells were included in the gating of pre-plasmablasts/plasmablasts (prePB/PB) as done by other groups (26, 46). CD138 expression has been linked to enhanced survival of ASC by promoting intrinsic survival cytokine signaling contributing to selection of more mature ASC in immune responses (59). Furthermore, CD138 was shown to bind APRIL on the surface of human multiple myeloma cells forming a survival loop (60). Given the fact that plasma cell development is impaired in newborns (12) and their survival is limited (4, 26) it is possible that low CD138 expression observed in neonatal mice is at least partly to blame. Additionally, diminished BCMA expression was observed in the pre-plasmablast/plasmablast population in contrast to plasmablasts/plasma cells (PB/PC), that had higher proportion of BCMA^+^ cells in neonates than adults (Figures 2C,E). This suggests that the few pre-plasmablasts/plasmablasts that are able to overcome the threshold of upregulating CD138 to a high level and differentiate into plasma cells retain their BCMA expression. LT-K63 enhanced the number and frequency of splenic plasmablasts, plasma cells and their BCMA expression (Figures 4A,B). BCMA binds both BAFF and APRIL, yet APRIL with a higher affinity. BCMA is selectively induced during plasma cell differentiation and promotes the survival of plasmablasts and long-lived plasma cells (11, 61). As for BAFF, LT-K63 prolonged APRIL secretion in spleen (Figure 4F) which is consistent with enhanced PPS1- and TT-specific ASC in spleen and TT-specific IgG Abs in serum 14 and 21 days after immunization (Figure 5). In this study there was only a trend for enhanced PPS-specific IgG Abs at these early time points (Figure 5C) in contrast to later time points in this study (Figure 8) and our previous results (35, 39).

It is crucial to assess various time points since effects of vaccine and adjuvants on particular cell populations can have different kinetics. For example, in mice immunized with Pnc1-TT+LT-K63, BAFF-R expression increased early (d4-8) (Figure 3A). Subsequently BCMA expression of pre-plasmablasts/plasmablasts in spleen increased (d8-21) (Figure 4A), preceding enhanced BCMA expression of plasmablasts/plasma cells (d14-21) (Figure 4B).

LT-K63 strongly enhances NF-κB expression in peritoneal macrophages 24 h after i.p. injection of adult mice (62). NF-κB is important for promoting transcription of *Tnfsrf13* (the gene that encodes APRIL) (63) and *Tnfsrf13b* (the gene that encodes BAFF) in macrophages (64). It is therefore possible that enhanced NF-κB translocation induced by LT-K63 directly affects the production of APRIL and BAFF that contribute to enhanced activation of B cells (Figures 3, 5) and their prolonged survival (Figure 8). This is in line with studies demonstrating that both ligands are essential for plasmablast survival (65).

After differentiation of B cells into plasmablasts/plasma cells in secondary lymphoid organs, these cells can relocate to the bone marrow, reside in specialized stromal niches and receive the necessary cytokine signals for survival and longevity (15, 17, 18). Most plasmablasts emerging from neonatal germinal centers home to the bone marrow, but cannot differentiate into long-lived plasma cells, and persist, due to lack of survival signals, such as APRIL. Instead they undergo apoptosis, causing transient Ab responses in early life (4, 5, 26). We demonstrated that LT-K63 increased vaccine-specific ASC and Abs (Figures 5, 8) as previously shown (33, 39, 45) and herein this increase was associated with enhanced plasmablasts and plasma cells and their BCMA expression in bone marrow (Figures 6, 8). Genetic knockout of *Tnfsrf17* (the gene that encodes BCMA) was shown to induce dramatic loss of bone marrow ASC in adult mice 6–8 weeks after immunization without affecting germinal center responses and early antigen specific Ab titers (11). This is in agreement with our results as retained BCMA expression 8 weeks after immunization with Pnc1-TT+LT-K63 coincided with enhanced vaccine-specific ASC and Abs in neonatal mice (Figure 8). Additionally, we have shown that immunization with Pnc1-TT+LT-K63 induces persistence of vaccine-specific Abs for at least 12 weeks (33).

We assessed, for the first time in neonates, accessory cells of the plasma cell survival niche and their APRIL secretion by flow cytometry. We showed that bone marrow eosinophils, megakaryocytes, and macrophages are increased by Pnc1-TT immunization with LT-K63, as well as their APRIL expression (Figure 7). This is in agreement with results from adult mice where eosinophils (25) and megakaryocytes (24) were shown to produce APRIL. Kinetics of the accessory cell accumulation following immunization differ; megakaryocyte frequencies in bone marrow increase early (d4) whereas eosinophil and macrophage frequencies increase later (d8-d21) (Figure 7). We note that the effect of LT-K63 on APRIL expression was very rapid since more than 60% of eosinophils, 40% of megakaryocytes, and 25% of macrophages were APRIL^+^ already 4 days after immunization. However, robust LT-K63-induced APRIL expression in the cells of the bone marrow niche declines rapidly to lower levels on day 14 than in mice immunized with Pnc1-TT only. This was unexpected since B cells need time to differentiate into plasmablasts/plasma cells and then relocate to the bone marrow. Nonetheless, APRIL has been shown to be retained in lung tissue by heparan sulfate proteoglycans (66, 67). It is therefore possible that in neonates, high levels of APRIL are secreted in the beginning of the immune response and are retained in the survival niche. We also note that APRIL expression of eosinophils is slightly higher in control mice receiving saline than Pnc1-TT 4 and 8 days after immunization, but much lower than in mice that received Pnc1-TT+LT-K63. Whether this reflects high constitutive APRIL expression or response to environmental triggers in this age group is unclear. Data on accessory cells of the bone marrow survival niche in neonates are limited where only Mac1^+^F4/80^+^Gr-1^−^ bone marrow resident macrophages were shown, by immunofluorescence staining, to increase plasmablast survival following immunization (26). However, it was not confirmed that those were the only cells with plasmablast-supporting capacity (26). These results are consistent with our findings, that not only macrophages, but also eosinophils and megakaryocytes express APRIL and can enhance early life plasma cell survival (Figure 7). We also assessed the frequency of monocytes and neutrophils and found that only a small proportion expressed APRIL (<5%), therefore they were not studied further. Prior to assessment of bone marrow accessory cells we depleted B, T, dendritic cells, basophils, and mast cells to better visualize small populations. However, T regulatory cells (68), dendritic cells (68, 69), and basophils (70) have all been shown to support plasma cell survival in adult mice and their relative contribution compared to eosinophils, macrophages, and megakaryocytes remains to be studied in neonatal mice. Two recent publications stating that eosinophils are not essential for plasma cell survival strengthen the theory of redundancies of accessory cells (71, 72).

Taken together, we have shown that the adjuvant LT-K63 enhances expression of BAFF-R on B cells, prolongs BAFF and APRIL expression in spleen leading to increased plasmablasts/plasma cells and their enhanced expression of BCMA. Furthermore, for the first time in neonatal mice, we found that LT-K63 enhanced the frequency and APRIL expression of eosinophils, macrophages, and megakaryocytes in bone marrow which likely promotes enhanced survival of plasma cells. This was all associated with enhanced, sustained vaccine-specific ASC in bone marrow and persistent vaccine-specific serum Abs. We have shed light on molecular mechanisms that should be triggered by vaccine adjuvants to induce robust and sustained immune responses to vaccination in early life. These newly defined markers of adjuvanticity represent parameters to be investigated in discovery and evaluation of potential adjuvants for the pediatric population and thus benefit the field of vaccine research and development.

## Data Availability Statement

The datasets generated for this study are available on request to the corresponding author.

## Ethics Statement

This study was performed in agreement with the Act on animal welfare (No. 55/2013) and regulations on protection of animals used for scientific research (No. 460/2017). The Experimental Animal Committee of Iceland approved the protocol (license no. 2015-10-01).

## Author Contributions

AA, IJ, and SB conceived and designed the study, analyzed the data, and wrote the manuscript. IJ and SB supervised the study. GD provided material and expertise. AA, JM, GM, and SB performed the experiments. AA, GD, IJ, and SB interpreted the results. All authors contributed to and approved the final version of the manuscript.

## Conflict of Interest

GD is full-time employee and holds shares in the GSK group of companies. The remaining authors declare that the research was conducted in the absence of any commercial or financial relationships that could be construed as a potential conflict of interest.
