# Supplementary material for: LT-K63 Enhances B Cell Activation and Survival Factors in Neonatal Mice That Translates Into Long-Lived Humoral Immunity
Source: Front Immunol. 2020 Oct 23;11:527310. doi: 10.3389/fimmu.2020.527310 (PMC7644473; doi:10.3389/fimmu.2020.527310)
Supplement: Supplementary file 1 [file Data_Sheet_1.doc]

Supplementary Material

# LT-K63 enhances B cell activation and survival factors in neonatal mice that translates into long-lived humoral immunity

Audur Anna Aradottir Pind^1,2^, Jenny Lorena Molina Estupiñan^1,2^, Gudbjorg Julia Magnusdottir^1,2^, Giuseppe Del Giudice^3^, Ingileif Jonsdottir^1,2^, Stefania P. Bjarnarson^1,2*^

^1^ Department of Immunology, Landspitali, the National University Hospital of Iceland, Reykjavik, Iceland;
^2^ Faculty of Medicine, School of Health Sciences, University of Iceland, Reykjavik, Iceland;

^3^ GSK Vaccines, Siena, Italy

*** Correspondance:**

Dr. Stefania P. Bjarnarson

stefbja@landspitali.is


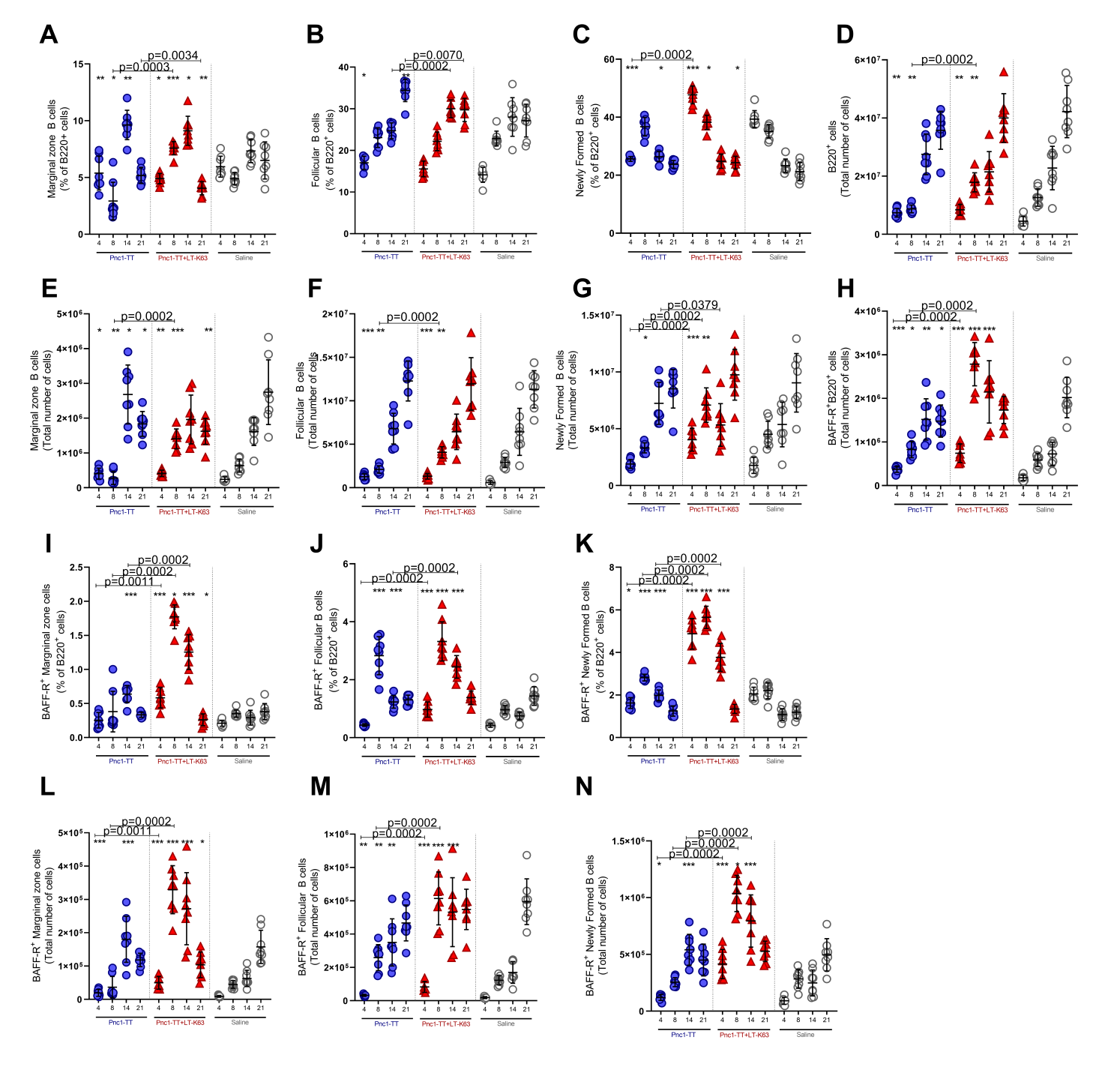


**Supplementary Figure 1.** Frequency of marginal zone (A), follicular (B) and newly formed B cells (C), total numbers of B220^+^ (D), marginal zone (E), follicular (F), newly formed B cells (G), BAFFR^+^ B220^+^ B cells, frequency of BAFFR^+^ marginal zone (I), follicular J) and newly formed B cells (K) and total number of BAFFR^+^ marginal zone (L), follicular (M) and newly formed B cells (N) following neonatal immunization with Pnc1-TT (blue), Pnc1-TT+LT-K63 (red) or saline (white). Each symbol represents one mouse and results are shown as means±SD for 8 mice per group per time point. For statistical evaluation Mann-Whitney U test was used. P-vaules are shown for the comparison of Pnc1-TT group to Pnc1-TT+LT-K63, stars represent comparisons of Pnc1-TT or Pnc1-TT+LT-K63 groups to saline group. *p≤0.05, **p≤0.01, ***p≤0.001.

**
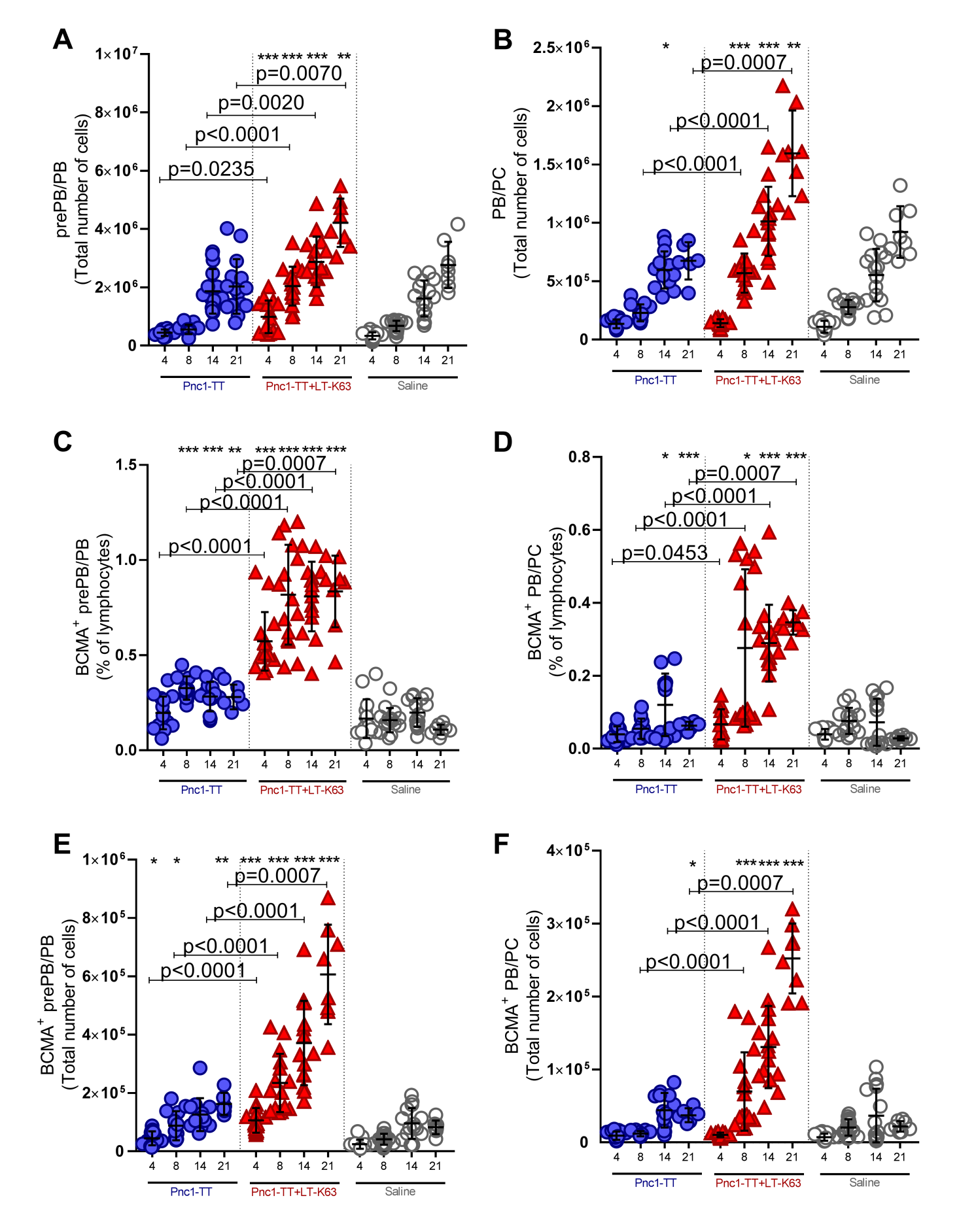

Supplementary Figure 2.** Total number of pre-plasmablasts/plasmablasts (prePB/PB; B220^+^C138^int^) (A) and plasmablasts/plasma cells (PB/PC; B220^+/-^CD138^high^) (B), frequency of BCMA^+^ prePB/PB cells (C) and BCMA^+^ PB/PC (D) and total number of BCMA^+^ prePB/PB (E) and BCMA^+^ PB/PC (F) in spleen assessed by flow cytometry 4, 8, 14 and 21 days after immunization of neonatal mice with Pnc1-TT (blue), Pnc1-TT+LT-K63 (red) or saline (white). Each symbol represents one mouse and results are shown as means±SD. Data is pooled from two independent experiments for days 4, 8 and 14 (n=7-8 for each experiment) but data for day 21 represents one experiment (n=8). For statistical evaluation Mann-Whitney U test was used. P-vaules are shown for the comparison of Pnc1-TT group to Pnc1-TT+LT-K63, stars represent comparisons of Pnc1-TT or Pnc1-TT+LT-K63 groups to saline group. *p≤0.05, **p≤0.01, ***p≤0.001.

#
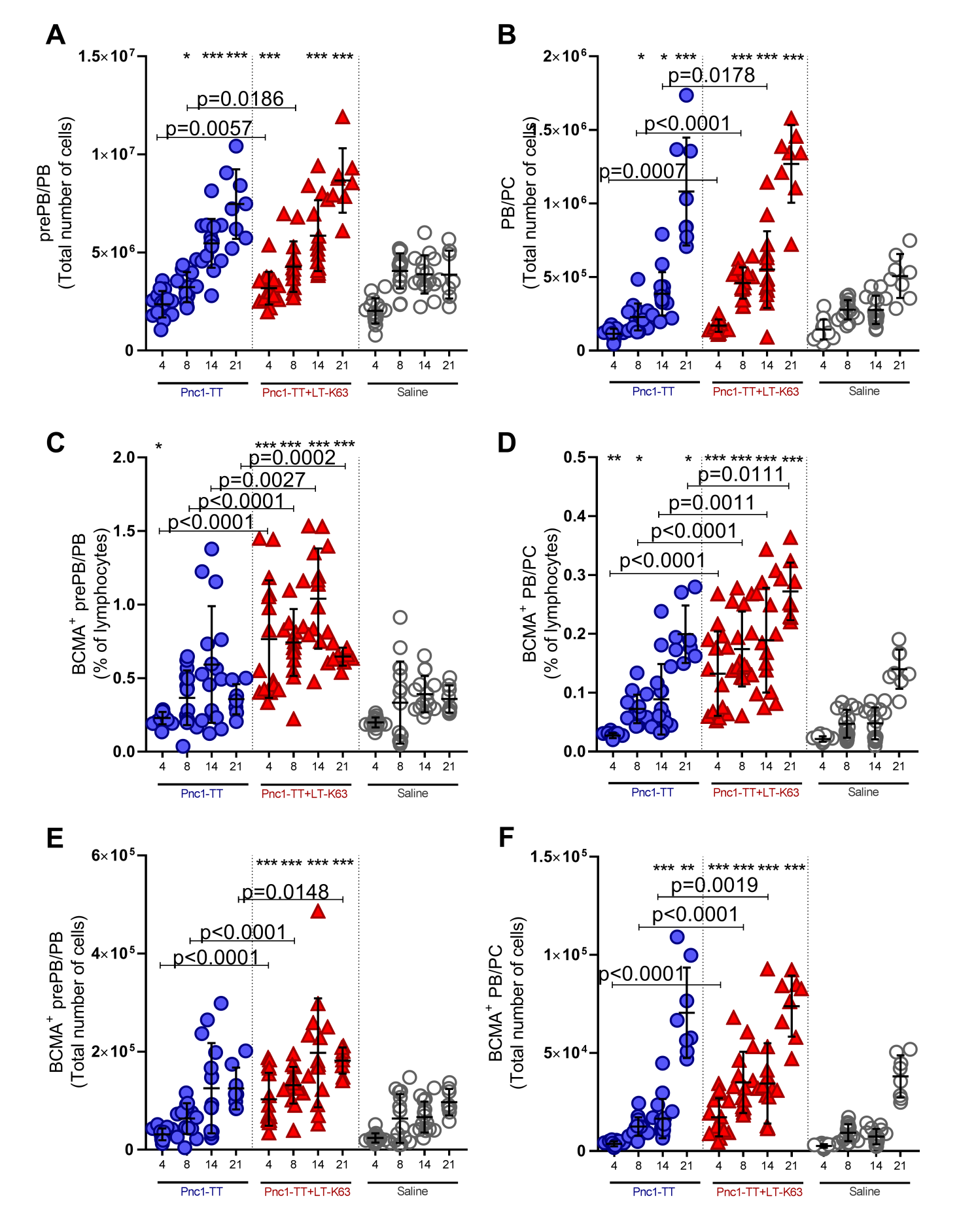


**Supplementary Figure 3.** Total number of pre-plasmablasts/plasmablasts (prePB/PB; B220^+^C138^int^) (A) and plasmablasts/plasma cells (PB/PC; B220^+/-^CD138^high^) (B), frequency of BCMA^+^ prePB/PB cells (C) and BCMA^+^ PB/PC (D) and total number of BCMA^+^ prePB/PB (E) and BCMA^+^ PB/PC (F) in bone marrow assessed by flow cytometry 4, 8, 14 and 21 days after immunization of neonatal mice with Pnc1-TT (blue), Pnc1-TT+LT-K63 (red) or saline (white). Each symbol represents one mouse and results are shown as means±SD. Data is pooled from two independent experiments for days 4, 8 and 14 (n=7-8 for each experiment) but data for day 21 represents one experiment (n=8). For statistical evaluation Mann-Whitney U test was used. P-vaules are shown for the comparison of Pnc1-TT group to Pnc1-TT+LT-K63, stars represent comparisons of Pnc1-TT or Pnc1-TT+LT-K63 groups to saline group. *p≤0.05, **p≤0.01, ***p≤0.001.

#
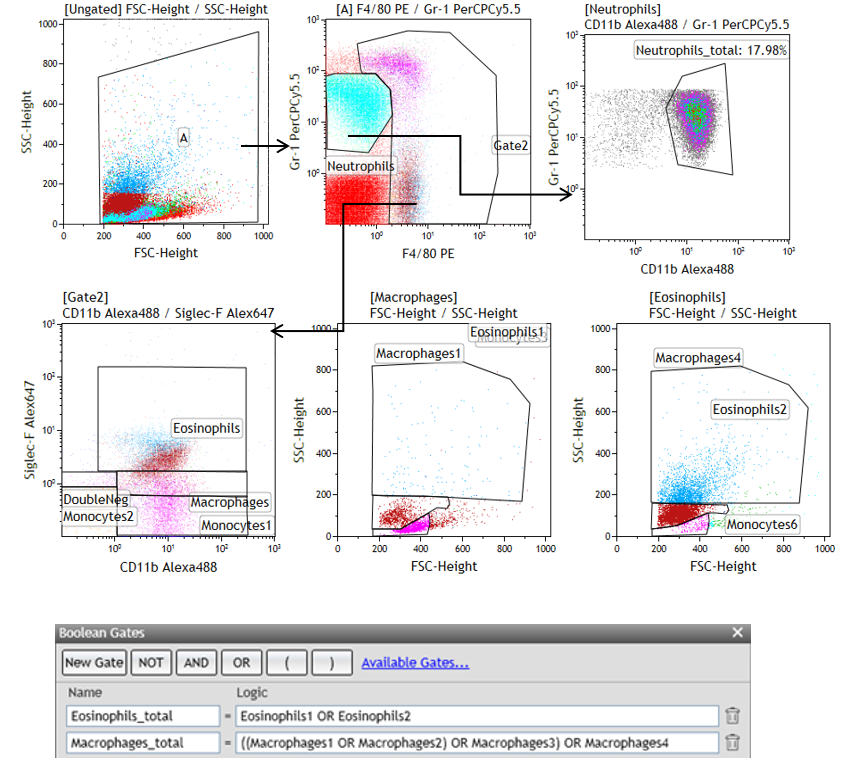


**Supplementary Figure 4.** Representative dot plots and gating strategy for eosinophils (Gr-1^int^F4/80^+^CD11b^+^Siglec-F^+^SSC^high^) and macrophages (Gr-1^int^F4/80^+^CD11b^+^Siglec-F^int^SSC^int^).

**
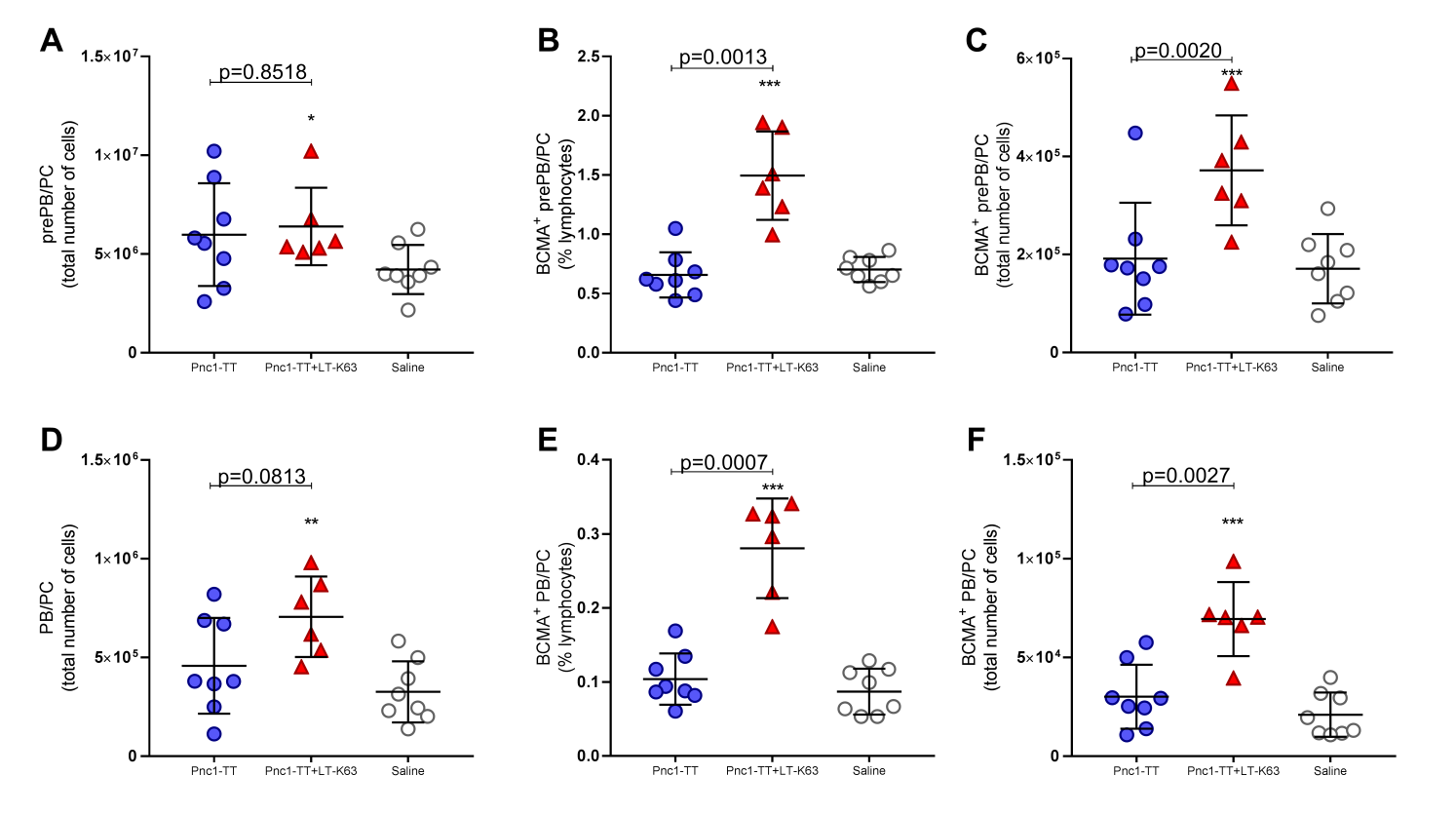
Supplementary Figure 5.** Total number of pre-plasmablasts/plasmablasts (prePB/PB; B220^+^C138^int^) (A) and frequency (B) and total number of BCMA^+^ prePB/PB (C), total number of plasmablasts/plasma cells (PB/PC; B220^+/-^CD138^high^) (D) and frequency (E) and total number of BCMA^+^ PB/PC (F) in bone marrow assessed by flow cytometry 8 weeks after immunization of neonatal mice with Pnc1-TT (blue), Pnc1-TT+LT-K63 (red) or saline (white). Each symbol represents one mouse and results are shown as means±SD for 6-8 mice per group. For statistical evaluation Mann-Whitney U test was used. P-vaules are shown for the comparison of Pnc1-TT group to Pnc1-TT+LT-K63, stars represent comparisons of Pnc1-TT or Pnc1-TT+LT-K63 groups to saline group. *p≤0.05, **p≤0.01, ***p≤0.001.

**
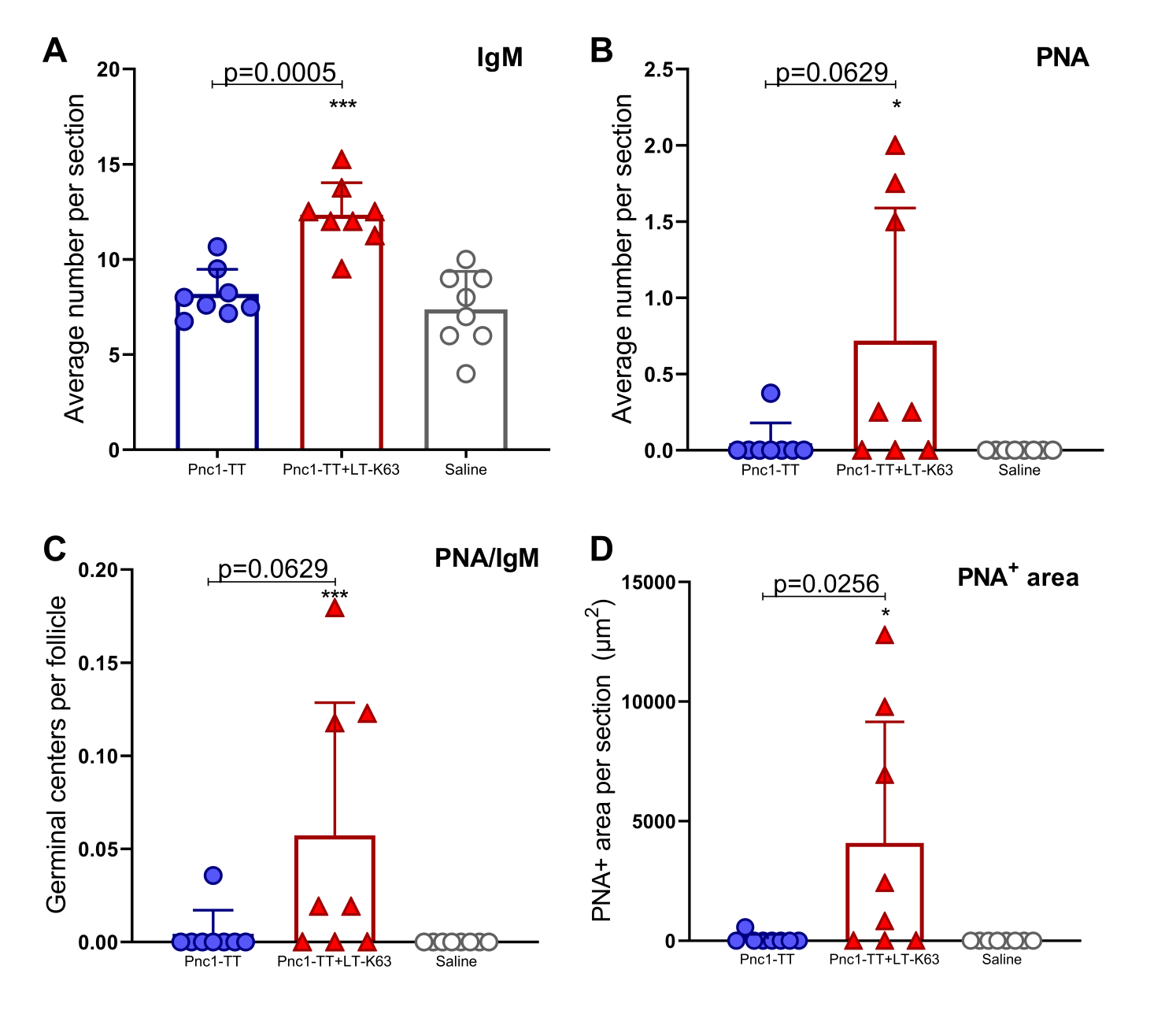
Supplementary Figure 6.** Spleen sections were stained with fluorescent antibodies for PNA and IgM 8 days after immunization of neonatal mice with Pnc1-TT (blue), Pnc1-TT+LT-K63 (red) or saline (white). IgM represents total number of follicles per section (A), PNA represents total number of activated follicles, germinal centers, per section (B), PNA/IgM ratio represents activated germinal centers in relation to total number of follicles (C) and PNA^+^ area represents total area of positive PNA staining per section. Each symbol represents one mouse and results are shown as means±SD for 8 mice per group. For statistical evaluation Mann-Whitney U test was used. P-vaules are shown for the comparison of Pnc1-TT group to Pnc1-TT+LT-K63, stars represent comparisons of Pnc1-TT or Pnc1-TT+LT-K63 groups to saline group. *p≤0.05, **p≤0.01, ***p≤0.001.
